# Comparison Between Spinal and General Anesthesia in Percutaneous Nephrolithotomy

**DOI:** 10.5812/aapm.13871

**Published:** 2013-12-26

**Authors:** Gholamreza Movasseghi, Valiollah Hassani, Mahmood Reza Mohaghegh, Reza Safaeian, Saeid Safari, Mohammad Mahdi Zamani, Roya Nabizadeh

**Affiliations:** 1 Department of Anesthesiology, Shahid Hamsheminejad Hospital, Iran University of Medical Sciences, Tehran, Iran; 2 Minimally Invasive Surgery Research Center, Iran University of Medical Sciences, Tehran, Iran; 3 Department of Anesthesiology and Critical Care, Iran University of Medical Sciences, Tehran, Iran

**Keywords:** Nephrostomy, Percutaneous, Hemodynamics, Analgesia, Hemorrhage

## Abstract

**Background:**

Hemodynamic stability and blood loss reduction are subjects to further consideration in patients undergoing percutaneous nephrolithotomy (PNCL).

**Objectives:**

This study compared the preference of spinal anaesthesia (SA) or general anaesthesia (GA) in respect to mentioned concerns.

**Patients and Methods:**

In this randomized clinical trial, 59 patients who underwent PCNL divided into SA and GA groups. 15-20 mg from intra-thecal bupivacaine 0.5%, and premedication of 0.01-0.02 mg from midazolam, were given to patients in SA group (n = 29). Patients in GA group (n = 30) received premedication of 1-2 µg/kg from fentanyl and 0.01-0.02 mg/kg from midazolam, and intravenously anaesthetized with 100 µg/kg/min of propofol and 0.5 mg/kg of atracurium, given by continuous infusion and N_2_O/O_2_ 50%. Mean arterial pressure (MAP) and heart rate were recorded intra-operatively and during recovery.

**Results:**

MAP and heart rate show no significant differences at designated time points between two groups (P > 0.05). Surgery time, anesthesia time, bleeding volume, and analgesic intake were significantly reduced in SA group (P < 0.05).

**Conclusions:**

It seems that, in patients undergoing PNCL, SA is as effective and safe as GA. Patients who undergo PNCL under SA require smaller amounts of analgesic dose and show hemodynamic stability during surgery and recovery time. Also, SA technique provides decreased blood loss and shortened surgery as well as anesthesia times compared to GA.

## 1. Background

Nowadays, percutaneous nephrolithotomy (PNCL) is a common method for extracting renal and urinary stones, and a choice modality in large, multiple, and stag-horn stones. Furthermore, PNCL can be used in patients with failed shock and endoscopic trials (1-3). In about 20% of cases, urologic procedures are undertaken with general anesthesia (GA) or regional anesthesia such as spinal anesthesia (SA). Despite good results of PNCL with GA, it may cause atelectasis, drug reactions, nausea, and vomiting (4, 5). In abdominal and lower extremities surgeries, SA is mainly employed by a single drug and comprises some advantages such as less bleeding, and reduces venous pressure in the surgery field (6, 7). However, there are recent reports regarding the use of SA in PNCL demonstrating lower post-operation pain, less drug intake, and reduced adverse effects. Some studies have also shown that surgeries with SA had better outcomes in spinal surgeries (4, 5, 8).

There are controversies among researchers regarding the use of SA in PNCL due to the most important issue which is acute hypotension, resulting from sympathetic block (9-12). Therefore, BP and pulse rate (PR) can be helpful to monitor sympathetic drive in these patients. There are many studies comparing GA and SA in several surgeries (13-17); however, there is no definite comparison made by BP and PR in PNCL during surgery and in recovery room. 

## 2. Objectives

Considering the type of anesthesia as well as patients' hemodynamics that can influence on surgery outcomes and relevant morbidity and mortality of the intervention, and that these factors directly reflect on regional health-care, we aimed this study to compare mean BP and PR among PNCL patients underwent GA and SA.

## 3. Patients and Methods 

### 3.1. Subjects

In this randomized clinical trial, all patients referred to Shahid Hasheminejad hospital in 2011 as PNCL candidates were included sequentially if they met these inclusion criteria: age between 18-65 years with physical status I or II of American Society of Anesthesiologists (ASA). All patients with spinal deformity, local infection at injection site, pregnancy, history of any neuromuscular or psychiatric disorder or chronic pain, who were suffering from hypertension, diabetes and coagulation disorders, patients with hypersensitivity to any anesthesia drugs, substance abusers, and patients who needed anesthesia higher than T4 and lower than T10 levels were excluded. The included patients were divided into SA and GA groups using randomized number table. Standard monitoring included continuous electrocardiogram, pulse oximetry, and end-tidal carbon dioxide. Noninvasive BP measurements were performed at 5-min intervals. All patients were routed with a green (18-gauge) catheter and infused with 3-4 cc/kg isotonic crystalloids. Maintenance venous liquid during surgery was based on 4/2/1 rule. For blood loss limited to "maximum allowable blood loss", 3 mL of Ringer solution was injected for every 1 mL of blood loss, and equal volume of matched iso-group packed cell for more blood losses. Both types of anesthesia were performed by a 4^th^ year resident of anesthesiology. 

### 3.2. GA Group

Premedication of 1-2 µg/kg from fentanyl and 0.01-0.02 mg/kg from midazolam was administered. Oxygen with an inspired fraction of 1.0 was administered for 3 min before intubation. Then, GA was induced by 3-5 mg/kg thiopental-Na, and to obtain desired anesthesia, 0.5 mg/kg of atracurium was injected intravenously for easier intubation; then, all patients were intubated by a suitable endotracheal tube. For maintaining GA, an intravenous 100 µg/kg/min of propofol with 50% O2 and 50% N2O were induced. The ventilation protocol consisted of an inspired oxygen fraction of 1.0, inspiratory to expiratory ratio of 1:2, and a respiratory rate adjusted to normocapnia (end-tidal carbon dioxide partial pressure between 30 and 40 mmHg). Mechanical ventilation has been set with a tidal volume of 10 ml/kg ideal body weight (IBW) and ZEEP (zero-positive end expiratory pressure). Atracurium and fentanyl re-administration was based on train-of-four (TOF) and every 45 minutes, respectively. 

### 3.3. SA Group

Premedication of 0.01-0.02 mg/kg from midazolam was administered. The patients were placed in a sitting position. The drug was administered by a 25-gauge Quincke needle in midline of L3-L4 or L4-L5 level by a physician. For inducing SA, isobar intra-thecal 15-20 mg of bupivacaine 0.5% without any additives was administered. Then, the patients' positions were changed to prone and intranasal 100% oxygen was administered. Sensory blockade was evaluated by a cotton peak (for heat perception) or a needle (for touching sense) every 15-20 seconds; then, motor blockade was tested by Bromage scale with following score: 0 = no paralysis; 1 = inability to raise extended leg; 2 = inability to flex knee; 3 = inability to move leg joints. Blood pressure below 100 mmHg of 30% from the baseline was corrected by 6 mg ephedrine and crystalloids, and all PR descents (less than 60/min) were treated by intravenous Atropine. All mentioned anesthetic drugs were provided by a regional pharmaceutical company (Darupakhsh, Iran).

### 3.4. Anesthesia Assessment

Systolic Blood Pressure (SBP), Diastolic Blood Pressure (DBP), Mean Arterial Pressure (MAP), and PR were recorded every 20 minutes during surgery from the beginning of anesthesia. Intraoperative blood loss was calculated by blood volume of suction devices, and estimated volume of blood in sponges and drapes already were weighted before operation. 

SBP, DBP, MAP, and PR were recorded in the PACU, every 10 min from entering PACU. Fifty mg from Meperidine was administered in patients suffered from additional pain. All patients were positioned in supine. MAP and PR were evaluated every 10 minutes for 1 hour. Other information were extracted from medical files and inserted into a pre-prepared checklist.

### 3.5. Ethical Issues

The patients were not charged by additional fees for the drugs used in any step of this study. The local ethics review committee of Iran University of medical sciences approved the study protocol. All participants gave written informed consent before participating.

### 3.6. Statistical Analysis

 Based on a pilot study in 12 patients (six from each group), we determined that a sample size of 26 in each group would be sufficient to detect the differences between mean of blood loss and analgesic demand, estimate a standard deviation of 10, a power of 95%, and a significance level of 5%; this number was increased to 30 per group, to allow a predicted drop-out of around 10% from the study.

The data were evaluated and analyzed by SPSS version 19 (SPSS Inc., Illinois, USA). All quantitative data were expressed as mean ± SD, and qualitative data as No. (%). For comparing the groups, t-test and Mann-Whitney-U test were used for parametric and non-parametric data, evaluated by Kolmogorov-Smirnov test, respectively. P less than 0.05 were considered as significant.

## 4. Results

### 4.1. Demographic Data

Fifty nine patients were enrolled in the study consisting of 38 males and 21 females. The patients were randomly divided into SA (n = 29) and GA (n = 30) groups. Table 1 demonstrates all demographic data. Surgery duration (P = 0.016) and anesthesia duration (P = 0.044) were significantly lower in SA (Table 2). According to Bromage scale, motor block level was zero in all patients in SA group. 

**Table 1.** Comparison of Demographics Between Two Groups

Variable	General Anesthesia	Spinal Anesthesia	P value
**Gender**			
Male, No. (%)	19 (63.3)	19 (65.5)	0.86
Female, No. (%)	11 (36.7)	10 (34.5)	
**ASA^a^Class**	22 (72.3)	23 (79.3)	0. 590
I			
II	8 (26.7)	6 (20.7)	
**Age, Mean ± SD, y**	46.9 ± 13.6	39.6 ± 9.7	0.022
**BMI^a^, Mean ± SD, kg/m^2^**	28.1 ± 4.6	26.4 ± 3.8	0.129

^a^Abbreviations: ASA, American Society of Anesthesiologists; BMI, body mass index

**Table 2.** Duration of Surgery, Anesthesia, Recovery time, Blood Loss, Analgesic Demand, and Blood Transfusion Amount in Both Groups

Variable	General Anesthesia	Spinal Anesthesia	P value
**Surgery Duration, Mean ± SD, min**	112.2 ± 18.3	99.3 ± 21.1	0.016
**Anesthesia Duration, Mean ± SD, min**	112.2 ± 18.3	101.3 ± 22.03	0.044
**Recovery Duration, Mean ± SD, min**	42.2 ± 12.8	41.5 ± 19.1	0.878
**Blood Loss, Mean ± SD, ml**	331.7 ± 151.1	211.03 ± 89.6	0.001
**Analgesicdemand, Mean ± SD**	6.3 ± 8.9	2.03 ± 6.3	0.038
**Blood Transfusion, No. (%)**			0.321
Positive	1 (3.3)	0 (0)	
Negative	29 (96.7)	29 (100)	

### 4.2. Endpoint Results

**Figure 1.**
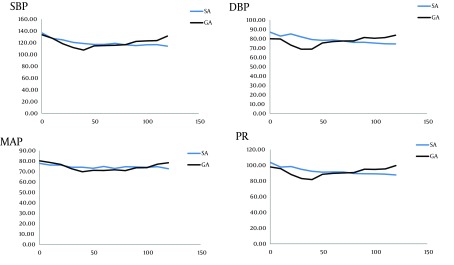
Trends of Systolic Blood Pressure (SBP), Diastolic Blood Pressure (DBP), Mean Arterial Pressure (MAP), and Pulse Rate (PR) in Operation Room. None of the factors differed significantly (P = 0.990, P = 0.568, P = 0.710, P = 0.934, respectively- from Repeated measurements)

In operation time-to-time analysis, SBP was significantly lower in GA group only in 120th minute; DBP in 60th, 90th, and 120th minutes, and MAP in 90th and 120th minutes (P < 0.05). The trend was not significantly different in none of 4 items (Figure 1; P > 0.05). 

**Figure 2.**
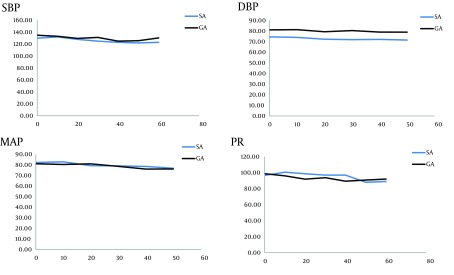
Trends of Systolic Blood Pressure (SBP), Diastolic Blood Pressure (DBP), Mean Arterial Pressure (MAP), and Pulse Rate (PR) in Recovery Room. None of the factors differed significantly (P = 0.844, P = 0.122, P = 0.863, P = 0.855, respectively- from Repeated measurements)

Table 2 demonstrates blood loss, analgesic demand, and blood transfusion amount in both groups. As seen, blood loss (P = 0.001) and analgesic demand (P = 0.038) were significantly higher in GA group. 

## 5. Discussion

Using SA in PNCL surgery is acceptable and more secure. By faster discharge and reduced recovery time, the patients’ quality of life can be improved using SA, which can be a good choice for urologist (18).

Overall, our study demonstrated that SBP, DBP, MAP, and PR in the whole surgery and recovery times did not have any significant difference between 2 groups, and that the trend was also somewhat similar in SA and GA; however, patients’ hemodynamics were more stable in SA group. Furthermore, bleeding and analgesic demand were significantly higher in GA group. None of the patients needed blood transfusion. These results were similar to other studies demonstrating that SA group had better hemodynamics and lower bleeding during and after the surgery (19-26).

In PACU, SBP was significantly lower in 10th, 20th, 30th, 40th minute; DBP and MAP in all evaluations and PR only in the 20th minutes were lower (P < 0.05). The trend was not significantly different in none of 4 items (Figure 2 ; P > 0.05).

It seems that SA can result in vasodilation and hypotension following sympathetic block. On the other hand, reduced intra-thoracic pressure and epidural vein distension, due to spontaneous ventilation, result in reduced bleeding. Therefore, the results do not seem to be irrational because SA can inhibit stress hormone secretion better than GA (27-30).

SA blocks preganglionic sympathetic nerves with many advantages compared to GA, such as redistribution of blood flow to musculoskeletal system, skin, and subcutaneous tissues, as well as reducing SBP, DBP, MAP, and PAP, and better hemostasis. Furthermore, other studies demonstrated better PNCL surgery results, lower blood loss, and lesser side effects (such as nausea, vomiting, and post-op pain) in SA (19, 31). Among these advantages of SA, decreasing blood loss is a main issue of SA in PCNL surgery. Recent studies investigated the effects of a 200-μg of oral clonidine tablet 60 - 90 minutes before anesthesia, which reduced blood loss significantly in several kinds of surgeries under GA that could be a future choice along with SA in PCNL (32, 33)

In McClain et al. study, SA could reduce the amount of anesthesia drugs, length of surgery time, and other side effects in discus decompression surgery (34). Tetzlaff et al. have also shown that in spinal surgeries, SA was a better choice for anesthesia compared to GA resulting in lower side effects (35). In an observational study, Mehrabi et al. evaluated 160 patients who underwent PCNL under spinal anesthesia in prone position. Blood transfusion was performed for ten patients (6.3%), and six patients complained of mild to moderate headache, dizziness, and mild postoperative low back pain for 2 to 4 days. Complete clearance of calculus or no significant residual calculi larger than 5 mm was achieved in 70% of patients (36). In another prospective randomized study on PCNL, 52 patients underwent general anesthesia and 58 patients received spinal anesthesia. PCNL was performed by standard technique. Intraoperative hypotension, postoperative headache, and low back pain were significantly higher in spinal group, but, compared to SA, the cost of anesthetic drugs was more than five times , and post-operative analgesic consumption about two times in GA group. Finally, authors suggested SA as a safe, effective, and cost-effective method in adult PCNL, the same as our results (37). Moreover , in other studies, additional analgesic consumption was reduced in SA group compared to GA group. This may be due to afferent nociceptive block of the spinal cord and faster block of sensory than that of motor nerves (13, 19).

In this study, patients with stone in upper pole of kidney, tolerated efficiently, but our sample size was designated for a whole kidney and not solely for upper pole; so because of general concerns about this subtype of kidney stones, future studies are needed with a study population designated for upper pole stones to compare competency and efficacy of SA versus GA.

In view of the results of our study, SA is a faster and safer method of anesthesia in PNCL surgeries. Using this method can help surgeons to maintain patient in a better hemodynamic and hemostatic state, reduce the GA complications, decrease the need of analgesics, and duration of surgery.
